# Immuno-Efficacy of a *T. gondii* Secreted Protein with an Altered Thrombospondin Repeat (TgSPATR) As a Novel DNA Vaccine Candidate against Acute Toxoplasmosis in BALB/c Mice

**DOI:** 10.3389/fmicb.2017.00216

**Published:** 2017-02-17

**Authors:** Bin Zheng, Jianzu Ding, Xiaoheng Chen, Haijie Yu, Di Lou, Qunbo Tong, Qingming Kong, Shaohong Lu

**Affiliations:** ^1^Immunology and Biochemistry Laboratory, Institute of Parasitic Diseases, Zhejiang Academy of Medical SciencesHangzhou, China; ^2^Jiaxing Vocational Technical CollegeJiaxing, China

**Keywords:** *Toxoplasma gondii*, toxoplasmosis, TgSPATR, vaccine, DNA plasmid

## Abstract

*Toxoplasma gondii* (*T*.gondii) is distributed worldwide and infects most species of warm-blooded animals, including humans. Toxoplasmosis has serious consequences, especially in people with an impaired or immature immune system. Thus, an effective vaccine is urgently required. Secretory microneme proteins are essential for the adhesion and invasion of *T. gondii*. The gene encoding the microneme protein, *T. gondii* secreted protein with an altered thrombospondin repeat (TgSPATR), we constructed a recombinant eukaryotic plasmid, pVAX1-TgSPATR, as a DNA vaccine, injected it intramuscularly into BALB/c mice and evaluated the induced immune response. Lymphocyte proliferation assays, cytokine (IFN-γ, IL-2, IL-4, IL-10), and antibody determinations showed that mice immunized with pVAX1-TgSPATR produced humoral and mixed Th1/Th2 type cellular immune responses. The survival times of mice immunized with pVAX1-TgSPATR were also significantly prolonged (15.7 ± 1.42 days) compared with control groups, which died within 7 days of challenge (*p* < 0.05). The current study indicated that pVAX1-TgSPATR induce a *T. gondii* specific immune response and might be a promising vaccine candidate against toxoplasmosis. To the best of our knowledge, this is the first report to evaluate the immunoprotective value of TgSPATR against *T. gondii*.

## Introduction

*Toxoplasma gondii*, of the phylum Apicomplexa, is an obligate intracellular parasite capable of infecting almost all species of warm-blooded animals, including humans (Lehmann et al., 2003; Piña-Vázquez et al., 2008). Although the primary infection is usually asymptomatic, toxoplasmosis still causes serious health issues for pregnant women, in which congenital infection can cause miscarriages, congenital diseases or other defects (Kravetz and Federman, 2005; Rorman et al., 2006). Additionally, toxoplasmosis can affect all types of livestock and causes serious economic losses to industry. Infected domestic animals are another route of transmission to humans (Dubey et al., 2005; Dautu et al., 2007; Zou et al., 2009). Thus, finding an effective vaccine against *T. gondii* is an important and realistic goal, which would be beneficial to both the humans and the farming industry.

In previous research, there were several kinds of vaccines have been tested their immunoprotective effects against toxoplasmosis, including attenuated vaccines, genetically engineered vaccines and DNA vaccines (Wang et al., 2007; Kur et al., 2009). Nonetheless, few of them have been authorized to use, mainly because of the low level of immune protection or biosafety issues (Innes and Vermeulen, 2006). Former study showed that inactivated vaccines could only provide moderate levels of protection. There is a commercial attenuated vaccine (ToxoVax®, Intervet B.V.) has been used in some areas, but the side effects as well as the expensive price all have limited the use of this vaccine (Mateus-Pinilla et al., 1999). In this context, we focus on the research of DNA vaccines, because these types of vaccines have been demonstrated to effectively induce both the long-term humoral and cellular immune responses against toxoplasmosis in animal models (Robinson, 1999; Gurunathan et al., 2000).

In recent years, great advancement has been made in the identification of vaccine candidates against *T. gondii* infection that could elicit a protective immune response (Jongert et al., 2008; Boothroyd, 2009).

In these potential vaccine antigens, *T. gondii* secreted protein with an altered thrombospondin repeat (TgSPATR) appears particularly promising. TgSPATR was a new member in microneme protein family, Ca^2+^-dependently secreted during early stage of invasion and existed on the outer surface of parasites (Kawase et al., 2010). This antigen is the homolog of *Plasmodium falciparum* SPATR (PfSPATR) which is immunogenic and recombinant SPATR antibodies could block sporozoite invasion (Chattopadhyay et al., 2003; Mahajan et al., 2005). Recent studies have shown that TgSPATR is contributed to *T. gondii* invasion and virulence. Δ*spatr* parasites were ~50% reduced in invasion compared to parental strains, a defect that was reversed in the complemented strain. In mouse virulence assays, Δ*spatr* parasites were significantly attenuated, with ~20% of mice surviving infection (Huynh et al., 2014). These findings demonstrate that TgSPATR may be a good vaccine candidate.

However, there have been no studies evaluating the potential of TgSPATR as a vaccine candidate against *T. gondii*. The purpose of this research was to detect the immunogenicity and protective value of TgSPATR in mice. We constructed a eukaryotic plasmid expressing TgSPATR and injected BALB/c mice intramuscularly to assess the immunoprotective effect of this DNA vaccine against the infection with the high virulence *T. gondii* RH strain in a BALB/c mouse model.

## Materials and methods

### Mice and parasites

Seven-week-old BALB/c mice were purchased from the Experimental Animal Center of Zhejiang Academy of Medical Sciences, China. All mice were maintained under standard conditions and the experimental procedures were in accordance with Chinese legislation on the use and care of laboratory animals.

Tachyzoites of the highly virulent *T. gondii* (type I) RH strain were maintained in our laboratory through serial passage in human foreskin fibroblast cells (HFF) grown in Dulbecco's modified Eagle's medium (DMEM) (Gibco, Carlsbad, CA, USA), supplemented with 5% fetal bovine serum (FBS) (Gibco, Carlsbad, CA, USA).

## Ethics statement

This study was carried out in strict accordance with the recommendations in the Guide for the Care and Use of Laboratory Animals of the National Institutes of Health. The protocol was approved by the Institutional Animal Care and Use Committee (IACUC) of Zhejiang Academy of Medical Sciences (Permit number: IACUC 2005-31).

### Construction of the eukaryotic expression plasmid

The TgSPATR open reading frame was amplified by polymerase chain reaction (PCR) from genomic DNA of *T. gondii* RH strain, using the following primers: sense primer : 5′-AAGCTTATGGAGGTTTCAAGAAGTCA-3′; antisense primer: 5′-GAATTCTTAAGACGAAGGCTGATTGC-3′, which introduce the *Hin*dIII and *Eco*RI restriction sites (underlined), respectively. The TgSPATR fragment was ligated into vector pVAX1 (Invitrogen, Carlsbad, CA, USA), digested with *Hin*dIII and *Eco*RI endonucleases, to construct the pVAX1-TgSPATR plasmid. To identify the positive plasmids, the recombinant plasmids were screened by PCR, double restriction enzyme digestion and DNA sequencing.

### Purification of pVAX1-TgSPATR and transfection cells

pVAX1-TgSPATR was purified from transformed *Escherichia coli* DH5α, using an Endofree plasmid giga kit (Qiagen, Chatsworth, CA, USA), dissolved in sterile endotoxin-free phosphate-buffered saline (PBS) and stored at −20°C. The DNA concentration was determined by absorbance at 260 nm using a NanoDrop 2000. Plasmid expression was analyzed by transfection of HEK293 cells using lipo2000 reagent (Invitrogen, Carlsbad, CA, USA), according to the manufacturer's instructions. After 2 d, cell monolayers and supernatants were collected and stored at −20°C. Expression of TgSPATR in the transfected cells was then analyzed by RT-PCR and western blotting.

### Western blotting analysis of TgSPATR

The cell lysates and purified rTgSPATR were separated by polyacrylamide gel electrophoresis and transferred onto PVDF membranes. Both cell lysates and purified rTgSPATR were probed with 1:100 dilutions of rabbit anti-*T. gondii* sera. Bound antibodies were detected using a horseradish peroxidase (HRP)-labeled goat anti-rabbit IgG, and the color was developed using tetra-methylbenzidine (TMB) (Sigma,St. Louis, MO, USA).

### Immunization schedule and challenge infection

Four groups of BALB/c mice (*n* = 15 each) were injected intramuscularly with 100 μg of pVAX1-TgSPATR plasmid DNA suspended in 100 μl sterile PBS in the thigh skeletal muscle (50 μl in each thigh skeletal muscle), whereas control groups mice received PBS, blank pVAX1 vector or nothing. Group I was injected with nothing as a blank control, group II with PBS (100 μl/each) alone as a control, group III with empty pVAX1 vector (100 μg/each) also as control, and group IV with pVAX1-TgSPATR. All groups were boosted with the same dose twice more with a 2-week interval.

Two weeks after the last booster injection, all the mice were challenged intraperitoneally with 100 tachyzoites of the virulent *T. gondii* RH strain. Blood was obtained from the mouse tail vein from mice in each group before each immunization at weeks 0, 2, and 4, and 2 weeks after the last booster (week 6). The serum was separated from the blood cells by centrifugation and stored at −20°C until used. Pre-immune serum samples were used as negative controls.

### Evaluation of humoral responses

Antigen-specific IgG antibodies were analyzed by ELISA using a standard procedure (Du and Wang, 2005). Briefly, 96-well micro-titer plates were coated overnight at 4°C with soluble tachyzoite antigens (STAg, 1 μg/well). The plates were washed three times with PBS containing 0.05% Tween-20 (PBST), pH 7.4. Nonspecific binding sites were blocked with PBS containing 10% BSA and incubated with mouse sera diluted 1:100 for 1 h at 37°C. After washing the plates in PBST, the bound antibodies were detected by HRP-conjugated goat anti-mouse IgG, IgG1, and IgG2a (Abcam, Cambridge, UK) diluted 1:10,000. Immune complexes were revealed with TMB as the substrate. The optical density (OD) was measured at 450 nm using an ELISA microplate reader (Bio-Rad 680, USA). For each serum sample, the assay was run in duplicate.

### Splenocyte proliferation by the MTT assay

Two weeks after the final booster injection, spleens were harvested from five mice in each group. Splenocyte suspensions were prepared and cultured in 96-well plates in triplicate at the density of 2 × 10^5^ cells/well in DMEM medium supplemented with FBS. Thereafter, the cultures were stimulated with either 10 μg/ml STAg, 5 μg/ml concanavalin A (Con A) (Sigma, St. Louis, MO, USA) as a positive control or medium alone as a negative control. The plates were incubated in 5% CO_2_ at 37°C for 68 h, before adding 50 μl of methyl thiazolyltetrazolium (MTT) (Sigma,St. Louis, MO, USA) solution to each well and incubating for 4 h. When the incubation was finished, 200 μl DMSO was added to each well before all the contents were discarded, and the absorbance was evaluated at 450 nm using an ELISA reader. The results were expressed as the stimulation index (SI), which is the ratio of the average OD_450_ value of the experimental samples containing antigen-stimulated cells to the average OD_450_ value of control cells cultured with medium alone.

### Cytokine analysis

To detect cytokines, spleen cells were obtained and cultured as described in the lymphocyte proliferation assay. The cell-free supernatants from cultured splenocytes were collected after 24, 72, and 96 h of different stimulations to analyze their interleukin-2 (IL-2) and interleukin-4 (IL-4), interleukin-10 (IL-10), gamma-interferon (IFN-γ) contents, respectively. The IL-2, IL-4, IL-10 and IFN-γ concentrations were evaluated using a commercial ELISA kit (eBioscience,San Diego, CA, USA) according to the manufacturer's instructions. All assays were performed in triplicate.

### Statistical analysis

Statistical analysis was carried out using one-way ANOVA for proliferation and cytokines production assays. The survival time was analyzed using the Kaplan-Meier method. Statistical analysis and graphics were carried out using SPSS software (SPSS Inc., Chicago, IL, USA). The results of the comparisons between groups were considered significantly different if *p* < 0.05.

## Results

### The expression of pVAX1-TgSPATR plasmid in HEK293 cells

**Figure 1:**
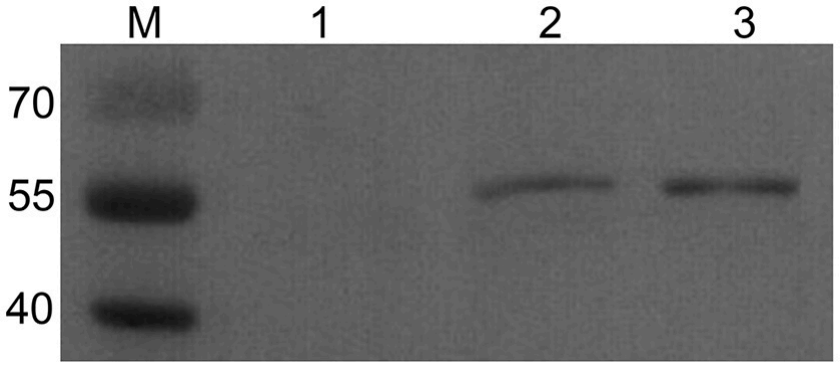
**Western blot analysis of TgSPATR expressed in HEK293**. M: marker 40–70 kDa; (1) negative control, the lysate of HEK293 transformed with empty pVAX1 vector; (2) the lysate of HEK293 transformed with pVAX1-TgSPATR; (3) purified rTgSPATR of *T. gondii* expressed in *E. coli*.

The TgSPATR gene was successfully amplified by PCR, ligated into the pVAX1 plasmid and produced in *E. coli* DH5α cells. In addition, western blotting analysis showed a specific band with a molecular mass of 55kDa, which is similar to the predicted mass in *T. gondii*, in HEK293 cells transformed with pVAX1-TgSPATR (Figure 1). The results demonstrated that the pVAX1-TgSPATR plasmid could express the target antigen in HEK293 cells.

### Specific IgG and IgG isotypes induced by vaccination

**Figure 2:**
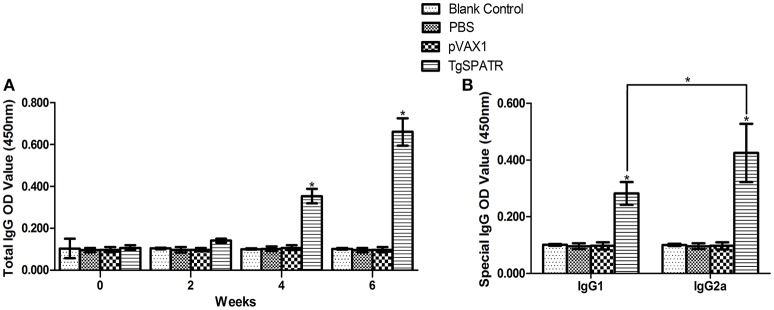
**Specific antibody responses induced by DNA immunization with pVAX1-TgSPATR, pVAX1, PBS and blank control, as assessed by ELISA. (A)** Determination of specific anti-TgSPATR antibodies in the sera of BALB/c mice. Serum samples were collected at 0, 2, 4, and 6 weeks post-primary immunization. **(B)** Determination of the specific anti-TgSPATR IgG subclass profile in the sera of immunized BALB/c mice. Results are expressed as means of the OD_450_ value and the standard deviation (*n* = 10); statistically significant differences (*p* < 0.05) are indicated by an asterisk (^*^).

To detect the levels of anti-*T. gondii* antibodies, the sera samples were tested by ELISA. The results are shown in (Figure 2A). The specific anti-TgSPATR antibodies titers were detectable as early as 2 weeks post-inoculation in the group immunized pVAX1-TgSPATR. During the test period, the level of the specific antibodies increased with successive immunizations and showed were significantly higher at the end of the test period compared with the other three former time points [*p* < 0.05; up to 0.66 ± 0.07 (mean ± S.D.)]. Mice injected with PBS or pVAX1 and blank control group did not generate significant antibody titers (*p* > 0.05).

Both IgG1 and IgG2a were found in the sera of mice immunized with pVAX1-TgSPATR and a relative increased ratio of IgG2a to IgG1 was observed (*p* < 0.05) (Figure 2B). No significant change was found in the control groups.

### *In vitro* splenocyte proliferation

The splenocytes from mice immunized with PBS, pVAX1, pVAX1-TgSPATR and blank control were prepared 2 weeks after the third immunization to assess the proliferative response. As shown in Table 1, the level of splenocyte proliferation in the group of mice injected with pVAX1-TgSPATR was higher than those of the other groups (*p* < 0.05); there was no remarkable change in the control groups. In addition, splenocytes from all experimental and control groups proliferated to comparable levels in response to the mitogen ConA (data not shown).

**Table 1:** **Cytokine production and the proliferative responses of splenocytes from BALB/c mice immunized with PBS, pVAX1, pVAX1-TgSPATR, or blank control**.

**Groups (*n* = 5)**	**Cytokine production (pg/ml)^a^**				**Proliferation(SI)^b^**
	**IFN-γ**	**IL-2**	**IL-4**	**IL-10**	
Blank control	44.23 ± 1.92	45.80 ± 1.73	48.99 ± 0.79	48.54 ± 0.12	0.64 ± 0.05
PBS	47.28 ± 2.34	43.43 ± 2.31	48.98 ± 3.28	49.36 ± 0.11	0.62 ± 0.01
pVAX1	44.11 ± 1.82	46.32 ± 3.26	49.19 ± 1.30	49.08 ± 0.03	0.55 ± 0.02
pVAX1-TgSPATR	672.87 ± 8.35^*^	367.93 ± 10.30^*^	212 ± 7.42^*^	261.8 ± 10.03^*^	1.24 ± 0.14^*^

a *Values for IL-2, IL-4, IL-10 and IFN- γ are for 24, 24, 72, and 96 h, respectively*.

b *SI stands for stimulation index. Splenocytes from mice were harvested 2 weeks after the last immunization. Data are presented as the mean ± S.D. (n = 5). Three independent experiments were performed*.

* *p < 0.05 compared with the control groups*.

### Cytokine production

The supernatants of splenocytes cultured from all groups of mice were harvested at different times after the re-stimulation with STAg and assessed for the production of cytokines of IFN-γ, IL-2, IL-4, and IL-10. Table 1 shows that splenocytes from mice immunized with pVAX1-TgSPATR secreted relatively large amounts of IFN-γ, IL-2, IL-4, and IL-10. In particular, significantly high levels of IFN-γ and IL-2 were detected in spleen cell cultures from mice injected with pVAX1-TgSPATR compared with groups immunized with the PBS, pVAX1 or the blank control (*p* < 0.05). Meanwhile, the levels of IL-4 and IL-10, which are Th2-type cytokines, were also significantly higher in the splenocytes of mice immunized with pVAX1-TgSPATR, compared with the other three groups (*p* < 0.05). The observation was correlated with the findings above that immunization with pVAX1-TgSPATR produced a mixed Th1/Th2 type response, which further confirmed the results of the IgG subclasses.

### Protective efficacy of DNA vaccine in mice against lethal toxoplasmosis

**Figure 3:**
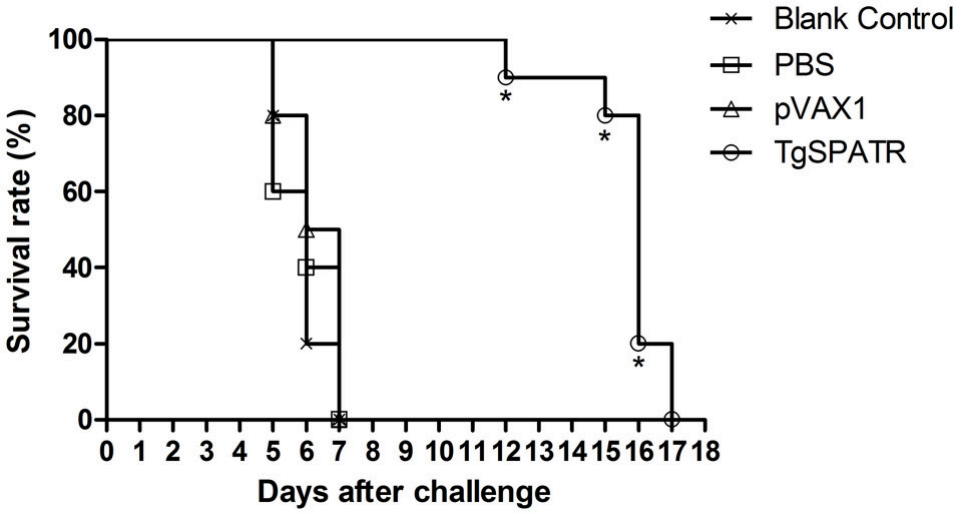
**Survival curves of BALB/c mice after challenge**. Survival curves of BALB/c mice injected with PBS, pVAX1, pVAX1-TgSPATR and the blank group, challenged with 100 tachyzoites of *T. gondii* RH strain 2 weeks after the last immunization. Each group comprised 10 mice. Statistically significant differences (*p* < 0.05) are indicated by an asterisk.

To determine whether the pVAX1-TgSPATR could induce a protective effect against *T. gondii* RH strain infection, immunized mice were challenged with 100 tachyzoites 2 weeks after the last immunization and their survival times were recorded (Figure 3). No difference was observed among the groups immunized with PBS, empty vector pVAX1 or blank control, all the mice in these three control groups died by the 7th day after infection. However, the mice immunized with pVAX1-TgSPATR showed an increased survival time (15.7 ± 1.42 days) compared with the control groups (*p* < 0.05).

## Discussion

DNA vaccinations have been shown to be a promising approach to protect animals and humans against pathogens, especially intracellular parasites, due to their low cost of production, thermal stability, and the ability to induce a wide range of cellular and humoral immune responses (Chen et al., 2013).

In the past few years, encouraging progress has been made in research into DNA vaccines against *T. gondii* infection. DNA-based plasmid vaccines could elicit humoral as well as cellular immunity and many have been developed and tested (Sharma and Khuller, 2001). In this study, we used the plasmid pVAX1™ as the vector, which contains appropriate genetic elements (e.g., promoter) required for *in vivo* expression of the antigen gene of interest in the target organism and meets U.S. Food and Drug Administra-tion (FDA) guidelines for design of DNA vaccines.

For instance, secretory microneme proteins are essential for adhesion and invasion during the attachment to the host cell surface (Beghetto et al., 2005). Using the MIC3 and MIC6 genes as DNA vaccines, the survival time of mice immunized with these DNA vaccines was significantly prolonged, while the control groups died in less than a week (Fang et al., 2009; Peng et al., 2009). In addition, ROP18 showed a promising immunoprotective value as a DNA vaccine. The survival time of mice immunized with pVAX1-ROP18 was significantly prolonged around 27.9 ± 15.1 days compared with the control groups, which were only survived 7 days after infection (Yuan et al., 2011). Another vaccine, pcDNA3.1-HisGRA6, helped to offer protective efficacy against toxoplasmosis and maintained 40% survival of BALB/c mice challenged with high virulence *T. gondii* RH strain (Sun et al., 2011). However, although these DNA vaccines presented a good protective effect against toxoplasmosis, they could not fully protect the mice from death. Thus, DNA vaccines with improved efficacy are required.

Thrombospondin-related anonymous protein (TRAP), one of the type 1 transmembrane protein, plays an important role in cell invasion and gliding motility by *Plasmodium* sporozoites (Sultan et al., 1997; Kappe et al., 1999). In the sporozoite stage, this kind of protein is expressed on the surface of cell and is associated with invasion into liver cells. This protein contains a Thrombospondin type 1 repeat domain (TSR), which is involved in cell attachment, mobility as well as extracellular protease activities (Mahajan et al., 2005). TSR domain appears in several kinds of surface proteins in *Plasmodium* spp., and proteins with this domain are important components of the invasion machinery. The thrombospondin-related anonymous proteins containing TSR domains are now mostly undergoing clinical trials as candidate vaccines (Alonso et al., 2004; Mahajan et al., 2005).

Several experiments showed that sera from clinically immune adults could recognize PfSPATR, while the serum from volunteers with low levels of anti-sporozoite antibodies and from control groups failed to recognize the antigen, which indicated that the host immune system could recognize PfSPATR and the reaction is specific. In addition, the anti-PfSPATR antibodies have been demonstrated to inhibit the sporozoite invasion of human liver cells, which also indicated the value of an SPATR vaccine (Menard et al., 1997; Chattopadhyay et al., 2003).

In *T. gondii*, TgSPATR also contains a TSR domain and it is a homolog of *Plasmodium* SPATRs; therefore, it might have similar functions to PfSPATR. Previous research found that TgSPATR was secreted and shed from the parasite surface at the early stage of invasion, in a Ca^2+^-dependent manner, as a new microneme protein, and played an important role in parasite adhesion and invasion (Nagamune et al., 2008; Kawase et al., 2010). Knocking out the TgSPATR gene reduced invasion by nearly 50% compared to parental strains, which was restored in the complemented strain. In the virulence assays of mouse model, Δ*spatr* parasites were significantly attenuated, with ~20% of mice surviving infection (Huynh et al., 2014). These observations prompted us to test TgSPATR as a potential candidate vaccine and drug target.

Therefore, we constructed the pVAX1-TgSPATR plasmid to test its immune protective effect. After the last boost, the mice immunized with pVAX1-TgSPATR had a relatively high level of splenocyte proliferation compared with control groups (*p* < 0.05). To characterizing the immune response, we chose IFN-γ, IL-2, IL-4, IL-10, and IgG2a and IgG1 isotypes as indicators of Th1 and Th2 responses. Both IgG2a (Th1) and IgG1 (Th2) were detected in immunized mice, with a slight increase in the anti-TgSPATR of IgG2a compared with IgG1 isotype (Figure 2B). In addition, the cellular immune response was associated with the production of IFN-γ and interleukin cytokines. From the cytokines results, a large amount of IFN-γ and IL-2 was produced. Meanwhile, the levels of IL-4 and IL-10, which are Th2-type cytokines, were also produced in significant amounts from the splenocytes of mice immunized with pVAX1-TgSPATR, compared with the three control groups (*p* < 0.05) (Table 1). These results indicated that pVAX1-TgSPATR triggered a mixed Th1/The2 type immune response. The high level of IFN-γ was similar to that noted in a previous study, where IFN-γ was the key cytokine in resistance against *T. gondii* infection (Dautu et al., 2007). The experimental results demonstrated that vaccination with pVAX1-TgSPATR was capable of enhancing the immune response in against *T. gondii* infection.

The survival rate of vaccinated mice against *T. gondii* challenge infection is considered the most direct consideration for assessing a candidate vaccine; therefore, we evaluated the protection by infecting the vaccinated mice intraperitoneally with tachyzoites of *T. gondii* RH. Although the mice did not survive after the infection, the mice immunized with pVAX1-TgSPATR did show a significantly prolonged survival time compared with those immunized with PBS, pVAX1 or blank control. The average survival time of the pVAX1-TgSPATR immunization group was 15.7 ± 1.42 days, while the control groups all died by the 7th day after infection (*p* < 0.05). The results indicated that the TgSPATR had the potential to be a new DNA immunization candidate. Ultimately, TgSPATR could be developed as a more effective vaccine by immunizing with the appropriate vaccine adjuvants, combining it with other effective DNA vaccines to produce multiantigenic DNA vaccines or using prime-boost strategy (Dziadek et al., 2011; Yuan et al., 2011; Quan et al., 2012).

In the present study, we found that immunization with TgSPATR DNA vaccine helps the mice survive longer significantly, but the mice were not capable of fighting high dosage of the lethal *T. gondii* parasite high virulent strain RH. However, our analysis may have been inappropriate for the assessment of protection. In future experiments, it would be helpful to validate the efficacy of *spatr* DNA vaccine immunization by comparing the survival rate in vaccinated and control groups using low virulence strains of *T. gondii*.

In conclusion, our study evaluated the immunogenicity and protective effect of a novel DNA vaccine expressing TgSPATR. Using the *T. gondii spatr* gene as a DNA vaccine, we were able to elicit a mixed Th1/Th2 type immune response, with high production of IFN-γ, IL-2, IL-4, and IL-10 in response to *T. gondii* infection. Moreover, the survival time increased significantly after the experimental challenge infection, all of which indicated that TgSPATR is a promising candidate vaccine against *T. gondii* infection.

## Author contributions

BZ, SL, XC, JD, HY, DL, QT, and QK developed the study protocol and data analysis. BZ, XC, JD, HY, and DL did the experiments. XC drafted the report. SL revised the report.

## Funding

This research is supported by National Natural Science Foundation of China (Grant No. 81572026), Zhejiang Science and Technology Project (2014C33230, 2015C37108), Science Foundation of Zhejiang Province of China (LQ15C180001, LQ17H190007) and Zhejiang Medical Science and Technology Plan project (2015KYB096).

### Conflict of interest statement

The authors declare that the research was conducted in the absence of any commercial or financial relationships that could be construed as a potential conflict of interest.
